# The Perception and Attitudes toward COVID-19 Vaccines: A Cross-Sectional Study in Poland

**DOI:** 10.3390/vaccines9040382

**Published:** 2021-04-14

**Authors:** Piotr Rzymski, Joanna Zeyland, Barbara Poniedziałek, Ilona Małecka, Jacek Wysocki

**Affiliations:** 1 Department of Environmental Medicine, Poznań University of Medical Sciences, 60-806 Poznań, Poland; bpon@ump.edu.pl; 2 Integrated Science Association (ISA), Universal Scientific Education and Research Network (USERN), 60-806 Poznań, Poland; 3 Department of Biochemistry and Biotechnology, Poznań University of Life Sciences, 60-632 Poznań, Poland; joanna.zeyland@up.poznan.pl; 4 Department of Preventive Medicine, Poznań University of Medical Sciences, 60-179 Poznań, Poland; imalecka@ump.edu.pl (I.M.); jawysocki@pro.onet.pl (J.W.)

**Keywords:** COVID-19, vaccinations, vaccine hesitancy, mRNA vaccines, vector vaccines, SARS-CoV-2

## Abstract

Vaccine hesitancy is a major threat to the success of COVID-19 vaccination programs. The present cross-sectional online survey of adult Poles (*n* = 1020) expressing a willingness to receive the COVID-19 vaccine was conducted between February and March 2021 and aimed to assess (i) the general trust in different types of vaccines, (ii) the level of acceptance of the COVID-19 vaccines already in use in Poland (BNT162b2 by BioNTech/Pfizer, mRNA-1273 by Moderna and AZD1222 by Oxford/AstraZeneca) as well as eight vaccines approved outside European Union (EU) or in advanced stages of clinical trials, (iii) level of fear of vaccination against COVID-19, and (iv) main sources of information on COVID-19 vaccination. Among all major vaccine technology, the highest level of trust was observed for the mRNA platform, with a considerable number of surveyed (>20%) not aware of the existence of vaccines produced using the traditional approach (inactivated and live attenuated vaccines). The age of participants was the main factor differentiating the level of trust in a particular vaccine type. Both BNT162b and mRNA-1273 received a high level of acceptance, contrary to AZD1222. From eight vaccines unauthorized in the EU at the moment of study, the CVnCoV (mRNA; CureVac) was met with the highest level of trust, followed by Ad26.COV2.S (vector; Janssen/Johnson&Johnson) and NVX-CoV2373 (protein; Novavax). Sputnik V (vector; Gamaleya Research Institute) was decidedly the least trusted vaccine. The median level of fear (measured by the 10-point Likert-type scale) in the studied group was 4.0, mostly related to the risk of serious allergic reactions, other severe adverse events and unknown long-term effects of vaccination. Female, individuals with a lower level of education and those not seeking any information on the COVID-19 vaccines revealed a higher fear of vaccination. Experts’ materials were the major source of information on COVID-19 vaccines in the studied group. The study shows the level of trust in COVID-19 vaccines can vary much across the producers while the mRNA vaccines are received with a high level of acceptance. It also emphasizes the need for effective and continuous science communication when fighting the pandemic as it may be an ideal time to increase the general awareness of vaccines.

## 1. Introduction

The COVID-19 pandemic has caused a challenge to healthcare systems, the economy, and education [1,2,3]. It put billions of people in quarantine during national lockdowns, magnifying pre-existing psychological and health issues and affecting various aspects of life [4,5]. By mid-March 2021, nearly 120 million cases of SARS-CoV-2 infection have been identified globally, with a death toll exceeding 2.6 million. Simultaneously, the health crisis has been counteracted by the scientific community’s unprecedented efforts encompassing basic research on SARS-CoV-2, epidemiological modeling, characterizing the clinical image of COVID-19, studies on repurposed drugs to treat the disease, and the development of vaccine candidates [6,7,8]. The unseen speed at which COVID-19 vaccines were made available is due to years of research and technological advances, the use of innovative platforms enabling rapid development of candidates, running multiple trials in parallel, significant funding, and help from regulatory institutions and their experts working at a higher pace [9,10]. In the European Union, the first vaccine, BNT162b2 by BioNTech/Pfizer, was authorized on 21 December 2020, followed by mRNA-1273 by Moderna and AZD1222 by Oxford/AstraZeneca approved on 7 and 29 January 2021, respectively. The third phase clinical trials of these vaccines have proven their efficacy in decreasing the number of symptomatic COVID-19 infections and disease severity [11,12,13]. Initial data originating from massive vaccinations reassure that these vaccines are an effective tool on the way to fight the pandemic [14].

However, one of the major threats to the COVID-19 vaccines rollout and successful mitigation of the pandemic is vaccine hesitancy [15]. There are several general factors influencing the reluctance to vaccination, including past experience with vaccines, level of education and knowledge, risk perception and trust, perceived importance of vaccination, subjective norms, and religious and moral convictions [16]. In the case of COVID-19 vaccines, additional factors may also play a role. Firstly, the speed at which the candidates were developed and approved within less than one year has raised some public concerns over their safety. Secondly, the number of questions regarding the durability of the immune response following the vaccination and vaccines’ effectiveness to limit the asymptomatic spread remained unanswered in the clinical trials [17]. Thirdly, the first COVID-19 vaccines’ approval was counteracted with an enormous range of scientifically unsupported claims, spread and amplified using online social media, potentially deteriorating the willingness to vaccinate among various groups of individuals [18,19,20,21]. Some studies also indicated the vaccine refusal within the healthcare workers, which is particularly problematic as it may impact the general public’s decision [22,23,24].

Moreover, differences in efficacy and a profile of solicited adverse effects may cause varying trust in particular COVID-19 vaccines. This may subsequently affect the willingness to vaccinate, particularly when people are not given a choice of vaccine they wish to receive. Last but not least, COVID-19 vaccination programs are conducted under extraordinary media attention and coverage. Therefore an acceptance level can be dynamically influenced by the quality of media content that a particular vaccine receives [25]. For this reason, the level of trust in COVID-19 vaccines must be monitored prior to and after their introduction in different world regions. Such information can help shape the strategies reaching out to the general public and support it in the decision-making process regarding the COVID-19 vaccination [26,27].

This study aimed to assess vaccines’ perception in Poland after two months since the first COVID-19 vaccines were introduced, while several others were under consideration or frequently discussed in media. Firstly, the general trust in vaccines based on their technology was evaluated in the surveyed group. This was followed by assessing the confidence in various COVID-19 vaccines authorized and unauthorized in the European Union. Finally, the level and main sources of fear related to vaccination and primary sources of information on COVID-19 vaccines were also investigated.

## 2. Materials and Methods

### 2.1. Survey

The anonymous, self-designed, and structured online questionnaire (Supplementary Materials) was made available through a media release by the Polish Press Agency (the single largest source of news in Poland) subsequently shared by the number of other traditional media outlets and their associated social media profiles, leading to the snowball effect. Such online research is a preferable approach to swiftly reach a group of individuals, ensuring their safety under pandemic conditions [28,29]. 

Specifically, the questionnaire employed in the study aimed to assess:

- The general level of trust in vaccines (using a 10-point Likert-type scale, where 1-no trust, 10-very high level of trust) in relation to their type: attenuated, inactivated, mRNA, vector-based, protein-based, and virus-like particles (VLPs). The mechanism of action of each vaccine was explained to the surveyed individuals using non-specialist language;
- The level of acceptance of the COVID-19 vaccines already approved and in use in Poland during the time of the study: BNT162b (BioNTech/Pfizer, Mainz/ Sandton, P O Box, Germany/USA), mRNA-1273 (Moderna, Cambridge, MA, USA) and AZD1222 (Oxford/AstraZeneca, Oxford, UK);
- The level of trust in other COVID-19 vaccines authorized outside the European Union or in advanced phases of clinical trials (using a 10-point Likert-type scale, where 1-no trust, 10-very high level of trust). These included vector vaccines Sputnik V (Gamaleya Research Institute, Moscow, Russia), Ad26.COV2.S (Janssen/Johnson&Johnson, Beerse/ New Brunswick, NJ, Belgium/USA), Ad5-nCoV (CanSino Biologics, Tianjin, China), mRNA vaccine CVnCoV (CureVac, Tübingen, Germany), protein vaccine NVX-CoV2373 (Novavax, Gaithersburg, MD, USA), inactivated vaccines CoronaVac (Sinovac Biotech, Beijing, China), BBIBP-CorV (Sinopharm, Beijing, China) and Covaxin (Bharat Biotech, Telagana, India);
- The level of fear prior to the vaccination against COVID-19 (using a 10-point Likert-type scale, where 1-no fear, 10-very high level of fear) and primary reasons behind this fear;
- The primary reasons behind the willingness to vaccinate against COVID-19;
- The primary sources of information on the COVID-19 vaccines in the surveyed group.

The demographic data on each surveyed individual included age, gender, place of living, and level of education. In addition, the data on whether the surveyed were identified as infected with SARS-CoV-2 and whether they lost the relative due to COVID-19 were also gathered.

The survey was conducted between 17 February and 11 March 2020, the day at which the European Medicines Agency (EMA) recommended the authorization of Ad26.COV2.S, developed by Janssen in co-operation with Johnson & Johnson. During the survey, three COVID-19 vaccines were already authorized in the European Union and used in Poland: BNT162b2 by BioNTech/Pfizer, mRNA-1273 by Moderna (both based on mRNA platform) and adenoviral-based vector AZD1222 (ChAdOx1-nCoV) developed by Oxford University in co-operation with AstraZeneca. At that time, the Polish COVID-19 vaccination program was mainly based on the BNT162b2 (given to healthcare workers and elderly) and AZD1222 (given primarily to kindergarten, school and academic teachers). During the survey period, 2,835,856 individuals in Poland (7.5% of the population) have received at least one dose of the COVID-19 vaccine. The inclusion criteria for the study were: no history of vaccination against COVID-19 but willingness to undertake it (confirmed with additional questions), Polish nationality, age ≥16 years old (a minimum age for which the use of COVID-19 vaccine, i.e., BNT162b2, was authorized in Poland at the time of the survey). The individuals with medical education were excluded from the analysis. 

Ten-point Likert-type scales to measure the level of trust in particular vaccines and fear of vaccination were selected based on literature review, which indicated that they were successfully applied in various cross-sectional studies investigating the vaccine acceptance and perceived risk of vaccination [30,31,32,33,34,35]. The questionnaire was pre-tested by the researchers, including the professional vaccinologist (J.W.). Considering the poll conducted in February that indicated that 55% of Poles are willing to receive the COVID-19 vaccine [36], the targeted population was approx. 16.5 million individuals. Following the survey completion, the representativeness of the sample size was calculated with Cochran’s formula [37]. A power calculation indicated that sample size (*n* = 1020) would give a margin error of 3.1% at the confidence level of 95%. The scales’ internal consistency reliability was determined with Cronbach’s alpha and showed good reliability of α = 0.87.

### 2.2. Statistical Analysis

The statistical analyses were performed using Statistica v.13.1 (StatSoft Inc., Tulsa, OK, USA). Because the level of trust and fear was measured by the ordinal Likert-type scale, non-parametric methods were applied. The difference between the two groups was analyzed with the Mann-Whitney U test. Differences in dichotomous data were evaluated by Pearson’s χ^2^ test. Bonferroni corrections were applied to any multiple comparisons to account for alpha inflation and limit the probability of type 1 error. A *p*-value < 0.05 was considered statistically significant and the exact values were reported in the text unless *p* < 0.001.

## 3. Results and Discussion

### 3.1. Demographic Characteristics

The survey was entered by 1206 individuals, of which 1020 met the inclusion criteria and were used in further analyses. The demographic breakdown of the studied group is presented in Table 1. Most of the surveyed were aged ≥50 (64.2%), female, inhabited urban areas (85.1%), predominantly cities >500 thousand inhabitants, and completed tertiary education (Table 1). The primary reasons behind the willingness to receive a COVID-19 vaccine included protecting oneself (64.2%) and relatives (60.6%) from infection, and putting an end to the pandemic (69.5%). In addition, a minority of surveyed (2.3%) indicated receiving a vaccine passport and related benefits (e.g., unrestricted traveling and better access to work opportunities) among the main reasons to get vaccinated. 

### 3.2. The General Trust in Vaccines in Relation to Their Type

**Figure 1:**
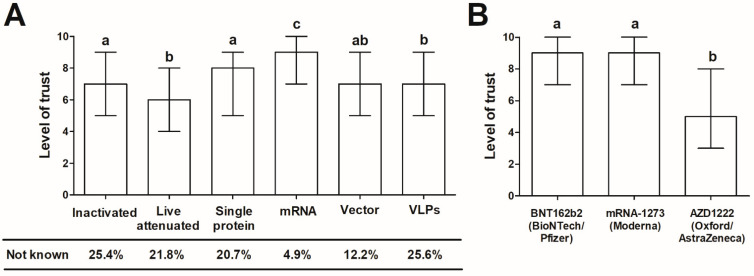
(**A**) The general level of trust (evaluated using 10-point Likert-type scale) in vaccines in relation to their technology and (**B**) trust in COVID-19 vaccines already approved and in use in Poland. The data is presented as the median and interquartile range. Different letters indicate statistically significant differences between vaccines (all *p* < 0.001, except (**A**) single protein vs. vector vaccine, for which *p* = 0.0013) demonstrated with the post-hoc Dunn’s test following the Kruskal-Wallis ANOVA (*p* < 0.001 for (**A**,**B**) comparisons).

The landscape of the COVID-19 vaccine candidates that emerged in 2020 has been highly diverse [38]. Beginning of September 2020, over 300 of them were under different stages of development and study [39], including candidates developed using a traditional approach (inactivated and attenuated vaccines) and modern solutions such as viral vectors, mRNA, single proteins, and virus-like particles as carriers. Considering that inactivated and live attenuated vaccines against human viral diseases have a relatively long history of use and are among the most successful preventive interventions [40,41], one could expect that they also are highly trusted. Contrary to this, the vaccines developed using the mRNA platform revealed the highest level of trust in the surveyed group. The mean level of vaccine acceptance in relation to the technology was as follows: mRNA > single protein > vector = virus-like particles = inactivated > live attenuated (Figure 1). 

However, a considerable number of surveyed admitted to being unaware of the existence of VLPs-based, inactivated, live attenuated and protein vaccines (25.6, 25.4, 21.8, and 20.7%, respectively), whereas only 4.9 and 12.2% reported not hearing of mRNA and vector vaccines, respectively. As shown, age and level of education were the only factors associated with awareness of particular vaccine types in the studied group. The individuals aged <50 and with a tertiary education were more frequently aware of each vaccine type except mRNA and vector vaccines that were not differentiated by the study participants’ age (Table 2).

As further shown, age was a significant factor differentiating the trust in a particular type of vaccine, with individuals <50 years revealing the higher level in all cases except mRNA, for which the high level of trust was noted in both age groups. The individuals with tertiary education more frequently indicated the level of trust >5 in the case of vector and VLP vaccines. Place of living and gender was not associated with the level of trust in any type of vaccine (Table 3). 

The present results highlight that the general public knowledge and awareness of vaccines in Poland is still not high, as already reported for various age groups in studies conducted prior to the COVID-19 pandemic [42]. Furthermore, the present research potentially indicates that temporary factors can drive the knowledge of vaccines. The COVID-19 vaccines based on mRNA were the first to be introduced in Poland and were met initially with numerous false claims, e.g. that their administration modifies the human genome, induces irreversible health damage, contains human immunodeficiency virus particles, or implants tracking chips. On the other hand, the educational materials on their mechanism of action and safety were prepared by or with help from experts and were continuously made available through different channels, including traditional and online media [26].

All in all, this highlights that a shift from the least trusted vaccine to the most accepted can be achieved in a short time if supported by substantial efforts from expert groups, national authorities, and media coverage. It also underscores a continued need to increase awareness of more traditional vaccines (such as inactivated and live attenuated), their mechanism of action and safety profile. These vaccines were already in use against other diseases long prior to the COVID-19 pandemic, although it is likely that knowledge of the technology under which they are developed is still poor and requires improvement using various channels. The present study clearly shows that age and, to less extent, education is related not only to awareness of the existence of the particular vaccine technologies but also the level of trust put in them. Therefore, the COVID-19 pandemic may be an ideal time to increase the general awareness of vaccines and their acceptance due to the public’s increased interest in vaccinations, an opportunity that should not be wasted. This is particularly important given the fact that in Poland, the percentage of parents who refuse immunization for their children has been growing in recent years [43], while the influenza coverage rate in the general population is very low (<5%) [44].

### 3.3. The Trust in COVID-19 Vaccines Authorized for Use in Poland

Among three COVID-19 vaccines approved for use in EU at the time of the study, both the BNT162b2 (BioNTech/Pfizer, Mainz/ Sandton, P O Box, Germany/USA) and mRNA-1273 (Moderna, Cambridge, MA, USA) vaccines gained a high level of trust in the surveyed group (Figure 1). The number individuals indicating a score ≥6 amounted to 84.1 and 82.3%, respectively. Contrary to this, the trust in AZD1222 (Oxford/AstraZeneca, Oxford, UK) vaccine was significantly lower, with 52.4% of surveyed indicating a score ≤5. The post-hoc comparisons clearly showed that AZD1222 is receiving a lower level of trust than BNT172b2 and mRNA-1273 (*p* < 0.001 in both cases, Dunn’s test following the Kruskal-Wallis ANOVA; Figure 1B). The level of trust in AZD1222 was also significantly lower than that put in vector-based vaccines (median (interquartile range) 5.0 (3.0–8.0) vs. 7.0 (5.0–9.0); *p* < 0.001, Mann-Whitney U test). Due to these observations, the potential factors differentiating trust in AZD1222 were further investigated. As found, it was higher in individuals with tertiary education as compared to those with lower levels of education (median (interquartile range) 6.0 (4.0–8.0) vs. 5.0 (3.0–7.0); *p* < 0.001, Mann-Whitney U test) and in those aged <50 compared to ≥50 (6.0 (4.0–8.0) vs. 5.0 (2.0–7.0; *p* < 0.001, Mann-Whitney U test), but did not vary between men and women, and urban and rural population (*p* > 0.05 in both cases, Mann-Whitney U test). 

There are several potential reasons behind such a poor acceptance of AZD1222, which likely act synergistically. Firstly, the third phase clinical trials of BNT162b2 and mRNA-1273 vaccines reported higher efficacy than the studies conducted for AZD1222 [11,12,13]. Although the direct comparison of these vaccines’ efficacy values is unfounded as they were studied separately and differed in timing, geographical regions, and dominating SARS-CoV-2 variants in circulation, these aspects are likely not apparent for the general public. Secondly, the mRNA vaccines were the first to be introduced in Poland and despite initial fears, no serious adverse effects were reported within the first weeks of administration.

Moreover, the mRNA vaccines received more coverage from media and expert groups regarding their mechanism of action, likely resulting in a higher level of understanding and acceptance. Thirdly, the difference may be due to varying awareness levels on adverse events following the vaccination between different groups of individuals. In Poland, AZD1222 was predominantly used to vaccinate teachers, contrary to elderly and healthcare workers vaccinated mostly with mRNA vaccines. The latter group is most informed on vaccines and the associated effects related to their administration. Notably, the pattern of frequency and severity of adverse events differs between mRNA and vector AZD1222 vaccines. According to the product information released by EMA, the adverse reactions are more likely following the first dose of AZD1222, while for the mRNA vaccines, they more often occur after the second dose. This, again, may not be well understood by the general public. As a result, the flu-like symptoms following the administration of the first dose of AZD1222 in school teachers received a high coverage by the Polish and European media leading to the false assumption that the AZD1222 vaccine is somewhat defective and less safe as compared to mRNA vaccines. Besides this, the AstraZeneca company has been receiving negative coverage in entire Europe due to reduced delivery of the vaccine doses, the cause of tensions in the European Union and searching for alternatives in vaccines unauthorized by EMA by selected political leaders [45]. Last but not least, during the present study, Denmark, Norway, and Iceland have temporarily suspended the rollout of the AZD1222 vaccine due to several cases of thromboembolic events. Due to similar reasons, Italy and Austria have stopped using specific batches of the drug as a precautionary measure [46]. After the completion of the present survey, the number of other European countries also temporarily suspended the vaccinations with AZD1222. Following the investigation by EMA that concluded that a vaccine is a safe and effective vaccine with benefits outweighing the possible risks, its rollout was set to be restarted in selected countries although at the moment of writing EMA’s analysis is still going [47]. 

Before the introduction of the first COVID-19 vaccines (BNT162b2 and mRNA-1273), both developed using mRNA platform) in Poland, there seem to be a high level of distrust toward them influenced by a massive spread of misinformation and scientifically unfounded claims regarding their mechanism of action and adverse effects of administration. This has been however counteracted by the expert activities with the help of mass media [26]. First polls, conducted in November 2020, demonstrated that only 20% of Poles declared a willingness to vaccinate. This increased to 36% in December 2020 and to 55% in mid-February 2021 [36,48]. The present study supports the notion that a strategy engaging experts and based on consistent, high-quality materials prepared in a manner accessible for non-specialists is pivotal in decreasing vaccine hesitancy. It also shows that temporary issues related to a particular vaccine may potentially affect the trust more significantly in individuals with lower education than tertiary, highlighting the continuous need for experts to be active in informing the general public and explaining peculiarities related to vaccine safety and efficacy that may be challenging to understand by individuals with no academic background.

The present study clearly shows that the perception of various authorized COVID-19 vaccines may differ significantly. It may also be prone to dynamic changes induced by a good or bad press that a particular vaccine receives. These differences should be taken into account when assigning the specific vaccine for use in the given group. Ensuring that those at very high risk of severe COVID-19 [49,50] will be offered the vaccine(s) with the greatest public trust level is pivotal in decreasing vaccine hesitancy and increasing vaccination rate in such priority groups.

### 3.4. The Trust in COVID-19 Vaccines Unauthorized in the European Union

The present study assessed the level of trust in eight COVID-19 vaccines unauthorized in the European Union and not used in Poland at the time of the study (Table 4). All of these vaccines were already in use in selected regions outside of the European Union or considered to be authorized (Ad26.COV2.S), and were all receiving some media coverage in Poland. The level of trust decreased in the following order: CVnCoV > Ad26.COV2.S = NVX-CoV2373 > CoronaVac = BBIBP-CorV = Covaxin = Ad5-nCoV > Sputnik V (Gamaleya Research Institute). However, one should note that over half of the surveyed have not heard of Chinese vaccines developed by the Sinovac Biotech, Sinopharm and Cansino Biologics, and Indian Covaxin by the Bharat Biotech. 

The highest level of trust in CVnCoV again indicates the acceptance of mRNA in the surveyed group of Poles which is likely due to the good reception of the already authorized BNT162b2 and mRNA-1273 vaccines, as also evidenced in this study. Due to decidedly low trust in the Sputnik V vaccine, the potential factors differentiating it were further investigated. As noted, it was significantly lower in women compared to men (median (interquartile range) 2.0 (1.0–5.0) vs. 3.0 (1.0–5.0); *p* = 0.008, Mann-Whitney U test). Other demographic factors (age, place of living, level of education) did not differentiate it (*p* > 0.05 in all cases, Mann-Whitney U test). There are several potential reasons behind the low level of trust in Sputnik V. Firstly, this vaccine was already approved in Russia in August 2020, three weeks before the first results from an open, non-randomized phase 1/2 clinical trials were published [51] and months before the interim data from the randomized, double-blind, placebo-controlled phase 3 were made available at the beginning of February 2021 [52], the time the vaccine was already in use numerous countries outside the EU. Such an approach, to authorize and then conduct the pivotal research, was met with a high level of disapproval from the scientific and medical community [53,54,55,56]. Secondly, Sputnik V’s distrust in the surveyed group is also likely to a generally high rate of unfavorable views of the Russian Federation in Poland, a result of a long and turbulent history of relations between these two countries [57]. Therefore, the potential low willingness to vaccinate with Sputnik V in Poland should be taken into account by the national health institutions if this vaccine will receive a positive recommendation from EMA [58]. 

### 3.5. Sources of Information on the COVID-19 Vaccines

**Figure 2:**
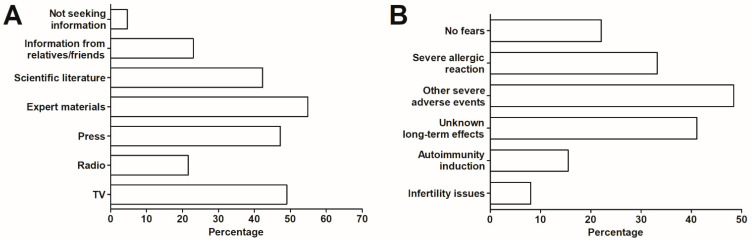
(**A**) The main sources of information regarding the COVID-19 vaccines and (**B**) primary reason of fear over the COVID-19 vaccination (*n* = 1020).

The materials prepared by expert groups were the primary source of information on the COVID-19 vaccines for the surveyed individuals, followed by the TV, press and scientific literature. Only 4.6% of surveyed admitted not to seek any information on the vaccines (Figure 2A)—this group was not differentiated by age, place of living, education, and gender from those who were actively interested in such information (*p* > 0.05 in all cases, Mann-Whitney U test).

These findings highlight the role of experts in the communication of science behind the vaccines developed and authorized during the pandemic. As described earlier, the number of such activities has been pursued in Poland and encompassed the publication of a White Paper on the COVID-19 vaccination, tracking and tackling emerging and circulating fake news, and engagement in Q&A sessions via different media channels [26]. Therefore, it is fully justified to initiate similar activities in other world regions, regardless of the current status and availability of the COVID-19 vaccines.

### 3.6. The Fears Related to COVID-19 Vaccination and Primary Reasons behind the Willingness to Receive a Vaccine

The general median (IQR) level of fear of the COVID-19 vaccine in the studied group (defined by the 10-point Likert-type scale) was 4.0 (2.0–5.0). Overall, 22.1% of surveyed individuals declared to have no fear regarding the vaccination. The main reasons for fear were related to the severity of adverse events (48.4%) and the onset of anaphylaxis or other serious allergic reaction (33.2%), as well as unknown long-term effects of the vaccine (41.1%) (Figure 2B). In addition, 2.2% indicated to worry that a vaccine will not protect them from the infection or that immunity will be short-lasting. 

A higher level of fear was noted in women then men (5.0 (2.0–5.0) vs. 3.0 (2.0–5.0), Mann-Whitney U test, *p* = 0.008), individuals with lower education level than tertiary (5.0 (3.0–6.0 vs. 4.0 (2.0–5.0), Mann-Whitney U test, *p* < 0.001), aged ≥50 than <50 (5.0 (3.0–6.0) vs. 4.0 (2.0–5.0, *p* < 0.001) and among those who did not seek any information on the COVID-19 vaccines compared to those who did (5.0 (4.0–7.0) vs. 4.0 (2.0–5.0), Mann-Whitney U test, *p* = 0.008). Being a convalescent patient, place of living and death to COVID-19 in the family (*p* > 0.05 in all cases, Mann-Whitney U test) did not differentiate the declared level of fear.

Prior to the study, it could be speculated that convalescent patients may express higher fear due to the assumption that their vaccination may lead to a higher frequency and more severe adverse events than in the general population. As observed in clinical trials of mRNA vaccines against COVID-19 [11,12], more frequent and severe adverse effects tended to be observed after the second dose, while for convalescent patients, the first dose represents their second exposure to an antigen. However, to date, there is no study that would clearly indicate that convalescent patients experience more severe or more frequent side effects after receiving the first dose. Nevertheless, the present study did not find the increased level of fear in convalescent patients. 

There are several potential explanations of higher fear reported by women. Firstly, substantial evidence indicates that women report a greater level of fear in general due to the number of gender differences [59]. Moreover, the previous studies have shown that women are more cautious in accepting innovative genetic-based technologies [60,61], while a variety of the COVID-19 vaccines are adopting them [62]. Finally, some false claims on COVID-19 vaccines are aimed explicitly at female health [63]. Compared to men surveyed in the present study, women expressed more frequently a fear over the vaccine’s effects on fertility (5.9 vs. 9.6%; *p* = 0.036, Pearson’s χ^2^ test). In addition, they also reported a fear over the induction of autoimmune disease (11.0 vs. 18.3%; *p* = 0.002, Pearson’s χ^2^ test) more commonly. 

It is well established that a lower level of education and knowledge of vaccinations is associated with vaccine hesitancy [64]. This again highlights experts and health professionals’ role in tailoring reliable information on COVID-19 vaccines using different channels for the highest possible outreach [26,27,65,66]. Their activities may increase the vaccination rate and improve the feeling of safety of those already willing to receive a vaccine. Subsequently, this may also positively affect the decision of individuals currently experiencing the COVID-19 vaccine hesitancy.

### 3.7. Limitations

Some study limitations should also be stressed out. Firstly, the research was based on an anonymous online survey that excludes the possibility of verifying the data on the more objective ground. Secondly, due to the short window to conduct the study, the applied questionnaire was only partially validated, with no pilot study ran on a subset of the intended population. Thirdly, an online survey may attract the attention and willingness of more young and better-educated individuals. Moreover, some subsets of surveyed were under-represented (e.g., convalescent patients), and therefore, the reported observations shall be treated with caution. One should also note that this study assessed only individuals expressing a will to receive a COVID-19 vaccine. At the time of the survey, approx. 55% of Poles were willing to do so [36]. Therefore, the present results should not be entirely extrapolated to the general population in Poland. Moreover, the willingness of the surveyed group to receive a COVID-19 vaccine may change over time due to varying factors (e.g., press coverage on the particular vaccine, authorization of subsequent vaccine candidates); thus, the results only represented a situation at the moment of study (mid-February to mid-March 2021). 

## 4. Conclusions

The study assessed the general trust in vaccines in the Polish population and the acceptance of the COVID-19 vaccines authorized and unauthorized in the European Union when the survey was conducted (late February–mid-March 2020). The results appear to support the notion that the battle against the COVID-19 pandemic is also ideal for increasing general awareness of vaccines. As revealed, vaccines developed using more modern technologies, such as mRNA platform, had a higher level of trust than the ones based on traditional approaches, with a relevant number of individuals not even recognizing the latter ones’ existence. The mRNA vaccines against COVID-19 also revealed the highest level of acceptance of all vaccines considered in the present study, indicating that if these vaccines become successful in fighting the pandemic, they may likely gain a general public acceptance for the mRNA platform as safe and effective tool to develop vaccines against other diseases. The observed differences in the level of trust in particular COVID-19 vaccines should be taken into account when considering their use in groups of the high risk of severe COVID-19. Recommending the one in which at the given moment is with the highest level of public trust will likely result in a better vaccination rate. The study also demonstrated the substantial role of expert’s activities in informing the general public on vaccinations. Such activities should be initiated or continuously pursued in various world regions to address vaccine hesitancy, tackle the false claims, and increase a willingness to vaccinate. 

## Figures and Tables

**Table 1:** The demographic characteristic of the studied group (*n* = 1020).

Age	Mean ± SD (Min–Max)	45 ± 15 (17–85)
<50	%/*n*	64.2 (655)
≥50		35.8 (365)
**Gender**	%/*n*	
Female		61.6 (628)
Male		38.4 (392)
**Place of living**	%/*n*	
Rural		14.9 (152)
Urban < 50,000		16.3 (166)
Urban 50,000–150,000		7.5 (77)
Urban 150,000–500,000		16.3 (166)
Urban > 500,000		45.0 (459)
**Education**	%/*n*	
Primary		0.2 (2)
Secondary		22.0 (222)
Vocational		3.8 (38)
Tertiary		74.0 (748)
**History of SARS-CoV-2 infection**	%/*n*	14.9 (152)
**Fatal case of COVID-19 in the family**	%/*n*	12.4 (126)

**Table 2:** The percentage of individuals in the studied group (*n* = 1020) aware of the particular vaccine type in relation to age, gender, education and place of living.

Vaccine Type	Age		χ^2^ *p*-Value	Gender		χ^2^ *p*-Value	Education		χ^2^ *p*-Value	Place of Living		χ^2^ *p*-Value
	<50	≥50		Male	Female		Tertiary	Other		Urban	Rural	
Inactivated	81.4	62.5	<0.001	73.2	76.8	ns	77.7	66.2	0.002	75.2	71.1	ns
Attenuated	84.3	67.4	<0.001	78.5	77.8	ns	81.8	68.4	<0.001	78.0	79.6	ns
Single protein	84.6	69.9	<0.001	78.5	80.6	ns	81.7	72.8	<0.001	79.1	80.3	ns
mRNA	95.6	94.3	ns	94.0	96.9	ns	96.0	92.7	0.011	94.9	96.1	ns
Vector	88.6	86.6	ns	85.8	91.0	ns	90.1	81.6	<0.001	87.9	87.5	ns
VLP	77.1	69.6	0.034	74.8	73.2	ns	78.7	62.5	<0.001	75.6	67.8	ns

ns—not significant.

**Table 3:** The percentage of individuals in the studied group (*n* = 1020) with the level of trust of >5 (in 1–10 Likert-like scale) in particular vaccine type in relation to age, gender, education and place of living.

Vaccine Type	Age		χ^2^ *p*-Value	Gender		χ^2^ *p*-Value	Education		χ^2^ *p*-Value	Place of Living		χ^2^ *p*-Value
	<50	≥50		Male	Female		Tertiary	Other		Urban	Rural	
Inactivated	76.9	41.2	<0.001	69.7	63.9	ns	66.9	63.8	ns	66.5	64.8	ns
Attenuated	64.1	30.5	<0.001	56.4	52.1	ns	55.1	49.5	ns	53.5	55.4	ns
Single protein	78.7	45.9	<0.001	71.8	66.1	ns	70.2	62.6	ns	69.0	64.7	ns
mRNA	85.1	81.4	ns	85.5	82.7	ns	84.5	81.7	ns	82.9	89.0	ns
Vector	65.6	52.5	<0.001	60.7	61.6	ns	64.2	51.3	0.002	61.5	58.6	ns
VLP	71.5	51.6	<0.001	65.4	64.5	ns	67.9	54.1	0.003	64.6	66.0	ns

ns—not significant.

**Table 4:** The level of trust (evaluated using 10-point Likert-type scale; median and interquartile range) in the COVID-19 vaccines not authorized in the European Union at the time of the study but in use in other world regions or in advanced stages of clinical trials (*n* = 1020).

Vaccine	Type	Manufacturer	Not Known [%]	Level of Trust
Sputnik V	Vector	Gamaleya Research Institute, Russia	17.0	2.0 (1.0–5.0) a
Ad26.COV2.S	Vector	Janssen/ Johnson&Johnson, The Netherlands/USA	13.9	5.0 (3.0–7.0) b
CVnCoV	mRNA	CureVac, Germany	42.7	7.0 (4.0–8.0) c
NVX-CoV2373	Single protein	Novavax, USA	49.1	5.0 (3.0–7.0) b
CoronaVac	Inactivated	Sinovac Biotech, China	55.4	4.0 (2.0–5.0) d
BBIBP-CorV	Inactivated	Sinopharm, China	57.0	5.0 (2.0–5.0) d
Covaxin	Inactivated	Bharat Biotech, India	63.2	5.0 (2.0–6.0) d
Ad5-nCoV	Vector	Cansino Biologics, China	63.3	4.0 (2.0–5.0) d

Different letters (a–d) indicate statistically significant differences between vaccines (all *p* < 0.001 except NVX-CoV2373 vs. CVnCoV for which *p* = 0.021). demonstrated with the post-hoc Dunn’s test following the Kruskal-Wallis ANOVA (*p* < 0.001).

## Data Availability

The data presented in this study are available from the corresponding author on reasonable request.

## Supplementary Materials

The following are available online at https://www.mdpi.com/article/10.3390/vaccines9040382/s1, Translated Questionnaire.

## References

[1] Lenzen M., Li M., Malik A., Pomponi F., Sun Y.-Y., Wiedmann T., Faturay F., Fry J., Gallego B., Geschke A. (2020). Global socio-economic losses and environmental gains from the Coronavirus pandemic. PLoS ONE.

[2] Betancourt J.A., Rosenberg M.A., Zevallos A., Brown J.R., Mileski M. (2020). The impact of COVID-19 on telemedicine utilization across multiple service lines in the United States. Healthcare.

[3] Rapanta C., Botturi L., Goodyear P., Guàrdia L., Koole M. (2020). Online University Teaching During and After the Covid-19 Crisis: Refocusing Teacher Presence and Learning Activity. Postdigit. Sci. Educ..

[4] Sidor A., Rzymski P. (2020). Dietary Choices and Habits during COVID-19 Lockdown: Experience from Poland. Nutrients.

[5] Prati G., Mancini A.D. (2021). The psychological impact of COVID-19 pandemic lockdowns: A review and meta-analysis of longitudinal studies and natural experiments. Psychol. Med..

[6] Nowakowska J., Sobocińska J., Lewicki M., Lemańska Ż., Rzymski P. (2020). When science goes viral: The research response during three months of the COVID-19 outbreak. Biomed. Pharmacother..

[7] Rzymski P., Nowicki M., Mullin G.E., Abraham A., Rodríguez-Román E., Petzold M.B., Bendau A., Sahu K.K., Ather A., Naviaux A.-F. (2020). Quantity does not equal quality: Scientific principles cannot be sacrificed. Int. Immunopharmacol..

[8] Gianola S., Jesus T.S., Bargeri S., Castellini G. (2020). Characteristics of academic publications, preprints, and registered clinical trials on the COVID-19 pandemic. PLoS ONE.

[9] Defendi H.G.T., da Silva Madeira L., Borschiver S. (2021). Analysis of the COVID-19 Vaccine Development Process: An Exploratory Study of Accelerating Factors and Innovative Environments. J. Pharm. Innov..

[10] Burgos R.M., Badowski M.E., Drwiega E., Ghassemi S., Griffith N., Herald F., Johnson M., Smith R.O., Michienzi S.M. (2021). The race to a COVID-19 vaccine: Opportunities and challenges in development and distribution. Drugs Context.

[11] Polack F.P., Thomas S.J., Kitchin N., Absalon J., Gurtman A., Lockhart S., Perez J.L., Pérez Marc G., Moreira E.D., Zerbini C. (2020). Safety and Efficacy of the BNT162b2 mRNA Covid-19 Vaccine. N. Engl. J. Med..

[12] Baden L.R., El Sahly H.M., Essink B., Kotloff K., Frey S., Novak R., Diemert D., Spector S.A., Rouphael N., Creech C.B. (2020). Efficacy and Safety of the mRNA-1273 SARS-CoV-2 Vaccine. N. Engl. J. Med..

[13] Voysey M., Clemens S.A.C., Madhi S.A., Weckx L.Y., Folegatti P.M., Aley P.K., Angus B., Baillie V.L., Barnabas S.L., Bhorat Q.E. (2021). Safety and efficacy of the ChAdOx1 nCoV-19 vaccine (AZD1222) against SARS-CoV-2: An interim analysis of four randomised controlled trials in Brazil, South Africa, and the UK. Lancet.

[14] Vasileiou E., Simpson C.R., Robertson C., Shi T., Kerr S., Agrawal U., Akbari A., Bedston S., Beggs J., Bradley D. (2021). Effectiveness of First Dose of COVID-19 Vaccines against Hospital Admissions in Scotland: National Prospective Cohort Study of 5.4 Million People. SSRN.

[15] Coustasse A., Kimble C., Maxik K. (2021). COVID-19 and Vaccine Hesitancy: A Challenge the United States Must Overcome. J. Ambul. Care Manag..

[16] Dubé E., Laberge C., Guay M., Bramadat P., Roy R., Bettinger J. (2013). Vaccine hesitancy: An overview. Hum. Vaccines Immunother..

[17] Baldo V., Reno C., Cocchio S., Fantini M.P. (2021). SARS-CoV-2/COVID-19 Vaccines: The Promises and the Challenges Ahead. Vaccines.

[18] Loomba S., de Figueiredo A., Piatek S.J., de Graaf K., Larson H.J. (2021). Measuring the impact of COVID-19 vaccine misinformation on vaccination intent in the UK and USA. Nat. Hum. Behav..

[19] Schiavo R. (2020). Vaccine communication in the age of COVID-19: Getting ready for an information war. J. Commun. Healthc..

[20] Johnson N.F., Velásquez N., Restrepo N.J., Leahy R., Gabriel N., El Oud S., Zheng M., Manrique P., Wuchty S., Lupu Y. (2020). The online competition between pro- and anti-vaccination views. Nature.

[21] Saied S.M., Saied E.M., Kabbash I.A., Abdo S.A.E.-F. (2021). Vaccine hesitancy: Beliefs and barriers associated with COVID-19 vaccination among Egyptian medical students. J. Med. Virol.

[22] Gagneux-Brunon A., Detoc M., Bruel S., Tardy B., Rozaire O., Frappe P., Botelho-Nevers E. (2020). Intention to get vaccinations against COVID-19 in French healthcare workers during the first pandemic wave: A cross sectional survey. J. Hosp. Infect..

[23] Gadoth A., Halbrook M., Martin-Blais R., Gray A., Tobin N.H., Ferbas K.G., Aldrovandi G.M., Rimoin A.W. (2020). Assessment of COVID-19 vaccine acceptance among healthcare workers in Los Angeles. medRxiv.

[24] Nzaji M.K., Ngombe L.K., Mwamba G.N., Ndala D.B.B., Miema J.M., Lungoyo C.L., Mwimba B.L., Bene A.C.M., Musenga E.M. (2020). Acceptability of Vaccination Against COVID-19 among Healthcare Workers in the Democratic Republic of the Congo. Pragmat Obs. Res..

[25] Boytchev H. (2021). Why did a German newspaper insist the Oxford AstraZeneca vaccine was inefficacious for older people—Without evidence?. BMJ.

[26] Rzymski P., Borkowski L., Drąg M., Flisiak R., Jemielity J., Krajewski J., Mastalerz-Migas A., Matyja A., Pyrć K., Simon K. (2021). The Strategies to Support the COVID-19 Vaccination with Evidence-Based Communication and Tackling Misinformation. Vaccines.

[27] Quinn S.C., Jamison A.M., Freimuth V. (2021). Communicating Effectively About Emergency Use Authorization and Vaccines in the COVID-19 Pandemic. Am. J. Public Health.

[28] Bazan D., Nowicki M., Rzymski P. (2021). Medical students as the volunteer workforce during the COVID-19 pandemic: Polish experience. Int. J. Disaster Risk Reduct..

[29] Geldsetzer P. (2020). Use of Rapid Online Surveys to Assess People’s Perceptions During Infectious Disease Outbreaks: A Cross-sectional Survey on COVID-19. J. Med. Internet Res..

[30] Albano L., Matuozzo A., Marinelli P., Di Giuseppe G. (2014). Knowledge, attitudes and behaviour of hospital health-care workers regarding influenza A/H1N1: A cross sectional survey. BMC Infect. Dis..

[31] Costantino C., Amodio E., Vitale F., Trucchi C., Maida C.M., Bono S.E., Caracci F., Sannasardo C.E., Scarpitta F., Vella C. (2020). Human Papilloma Virus Infection and Vaccination: Pre-Post Intervention Analysis on Knowledge, Attitudes and Willingness to Vaccinate Among Preadolescents Attending Secondary Schools of Palermo, Sicily. Int. J. Environ. Res. Public Health.

[32] Dubé E., Gagnon D., Ouakki M., Bettinger J.A., Guay M., Halperin S., Wilson K., Graham J., Witteman H.O., MacDonald S. (2016). Understanding Vaccine Hesitancy in Canada: Results of a Consultation Study by the Canadian Immunization Research Network. PLoS ONE.

[33] Di Giuseppe G., Abbate R., Liguori G., Albano L., Angelillo I.F. (2008). Human papillomavirus and vaccination: Knowledge, attitudes, and behavioural intention in adolescents and young women in Italy. Br. J. Cancer.

[34] Napolitano F., Napolitano P., Angelillo I.F. (2017). Seasonal influenza vaccination in pregnant women: Knowledge, attitudes, and behaviors in Italy. BMC Infect. Dis..

[35] Alholm Z., Ault K., Zwick R., Fitzgerald S., Satterwhite C. (2017). Pregnant Women’s Acceptance of Hypothetical Zika Vaccine. Open Forum Infect. Dis..

[36] CBOS Research Reports. https://www.cbos.pl/EN/publications/reports.php.

[37] Cochran W.G. (1977). Sampling Techniques.

[38] Le T.T., Cramer J.P., Chen R., Mayhew S. (2020). Evolution of the COVID-19 vaccine development landscape. Nat. Rev. Drug Discov..

[39] Forni G., Mantovani A., Forni G., Mantovani A., Moretta L., Rappuoli R., Rezza G., Bagnasco A., Barsacchi G., Bussolati G. (2021). COVID-19 vaccines: Where we stand and challenges ahead. Cell Death Differ..

[40] Plotkin S. (2014). History of vaccination. Proc. Natl. Acad. Sci. USA.

[41] Minor P.D. (2015). Live attenuated vaccines: Historical successes and current challenges. Virology.

[42] Zarobkiewicz M.K., Zimecka A., Zuzak T., Cieślak D., Roliński J., Grywalska E. (2017). Vaccination among Polish university students. Knowledge, beliefs and anti-vaccination attitudes. Hum. Vaccines Immunother..

[43] Kraśnicka J., Krajewska-Kułak E., Klimaszewska K., Cybulski M., Guzowski A., Kowalewska B., Jankowiak B., Rolka H., Doroszkiewicz H., Kułak W. (2018). Mandatory and recommended vaccinations in Poland in the views of parents. Hum. Vaccines Immunother..

[44] Nitsch-Osuch A., Gołębiak I., Wyszkowska D., Rosińska R., Kargul L., Szuba B., Tyszko P., Brydak L.B. (2017). Influenza Vaccination Coverage Among Polish Patients with Chronic Diseases. Adv. Exp. Med. Biol..

[45] Kar-Gupta S. EU Must Be United over Russian, Chinese COVID-19 Vaccines: French Minister. https://www.reuters.com/article/us-health-coronavirus-eu-france-idUSKBN2AX0NF.

[46] Wise J. (2021). Covid-19: European countries suspend use of Oxford-AstraZeneca vaccine after reports of blood clots. BMJ.

[47] Ring S., Fourcade M. Europe Restarts AstraZeneca Vaccines after Safety Endorsement. https://www.bloomberg.com/news/articles/2021-03-18/italy-france-to-restart-astra-vaccines-after-safety-endorsement.

[48] CBOS Attitudes to Vaccination against Covid-19. https://www.cbos.pl/EN/publications/reports/2020/154_20.pdf.

[49] Persad G., Peek M.E., Emanuel E.J. (2020). Fairly Prioritizing Groups for Access to COVID-19 Vaccines. JAMA.

[50] Ribas A., Sengupta R., Locke T., Zaidi S.K., Campbell K.M., Carethers J.M., Jaffee E.M., Wherry E.J., Soria J.-C., D’Souza G. (2021). Priority COVID-19 Vaccination for Patients with Cancer while Vaccine Supply Is Limited. Cancer Discov..

[51] Logunov D.Y., Dolzhikova I.V., Zubkova O.V., Tukhvatulin A.I., Shcheblyakov D.V., Dzharullaeva A.S., Grousova D.M., Erokhova A.S., Kovyrshina A.V., Botikov A.G. (2020). Safety and immunogenicity of an rAd26 and rAd5 vector-based heterologous prime-boost COVID-19 vaccine in two formulations: Two open, non-randomised phase 1/2 studies from Russia. Lancet.

[52] Logunov D.Y., Dolzhikova I.V., Shcheblyakov D.V., Tukhvatulin A.I., Zubkova O.V., Dzharullaeva A.S., Kovyrshina A.V., Lubenets N.L., Grousova D.M., Erokhova A.S. (2021). Safety and efficacy of an rAd26 and rAd5 vector-based heterologous prime-boost COVID-19 vaccine: An interim analysis of a randomised controlled phase 3 trial in Russia. Lancet.

[53] Bucci E., Andreev K., Björkman A., Calogero R.A., Carafoli E., Carninci P., Castagnoli P., Cossarizza A., Mussini C., Guerin P. (2020). Safety and efficacy of the Russian COVID-19 vaccine: More information needed. Lancet.

[54] Burki T.K. (2020). The Russian vaccine for COVID-19. Lancet Respir. Med..

[55] Balakrishnan V.S. (2020). The arrival of Sputnik V. Lancet Infect. Dis..

[56] Thorp H.H. (2020). A dangerous rush for vaccines. Science.

[57] Pew Research Center Russia’s Global Image Negative amid Crisis in Ukraine. https://www.pewresearch.org/global/2014/07/09/russias-global-image-negative-amid-crisis-in-ukraine/.

[58] European Medicines Agency EMA Starts Rolling Review of the Sputnik V COVID-19 Vaccine. https://www.ema.europa.eu/en/news/ema-starts-rolling-review-sputnik-v-covid-19-vaccine.

[59] McLean C.P., Anderson E.R. (2009). Brave men and timid women? A review of the gender differences in fear and anxiety. Clin. Psychol. Rev..

[60] Napolitano C.L., Ogunseitan O.A. (1999). Gender Differences in the Perception of Genetic Engineering Applied to Human Reproduction. Soc. Indic. Res..

[61] Simon R.M. (2010). Gender differences in knowledge and attitude towards biotechnology. Public Underst. Sci..

[62] Dolgin E. (2021). How COVID unlocked the power of RNA vaccines. Nature.

[63] Male V. (2021). Are COVID-19 vaccines safe in pregnancy?. Nat. Rev. Immunol..

[64] Ferdinand K.C., Nedunchezhian S., Reddy T.K. (2020). The COVID-19 and Influenza “Twindemic”: Barriers to Influenza Vaccination and Potential Acceptance of SARS-CoV2 Vaccination in African Americans. J. Natl. Med. Assoc..

[65] Brownlie J., Howson A. (2006). ‘Between the demands of truth and government’: Health practitioners, trust and immunisation work. Soc. Sci. Med..

[66] Larson H.J., Cooper L.Z., Eskola J., Katz S.L., Ratzan S. (2011). Addressing the vaccine confidence gap. Lancet.

