# Supplementary material for: The Perception and Attitudes toward COVID-19 Vaccines: A Cross-Sectional Study in Poland
Source: Vaccines (Basel). 2021 Apr 14;9(4):382. doi: 10.3390/vaccines9040382 (PMC8069794; doi:10.3390/vaccines9040382)
Supplement: Supplementary file 1 [file vaccines-09-00382-s001.pdf]

TRANSLATED QUESTIONNAIRE

1. **Have you been vaccinated against COVID-19?** Yes No
2. **Do you wish to receive the vaccine against COVID-19?** Yes No
3. **Please evaluate your level of trust in the vaccines in relation to their type using a 1-10 scale, where 0 – I have not heard about this vaccine type, 1 – no trust, 5- medium level of trust, 10 – high level of trust.**

**Vaccine type**	0	1	2	3	4	5	6	7	8	9	10
**Killed (inactivated) vaccines.** *Contain the pathogen which has been killed using heat, chemicals, or radiation.*											
**Live (attenuated) vaccines.** *Contain a weakened form of the pathogen that does not cause severe disease.*											
**Protein vaccines.** *Contain purified protein of the pathogen obtained from modified cells that can produce it.*											
**mRNA vaccines.** *Contain mRNA particles that provide instruction for the cell on how to make a piece of pathogen's protein.*											
**Vector vaccines.** *Contain viruses that genomes contain a gene instructing the cells how to make a piece of pathogen's protein.*											
**Virus-like particles vaccine.** *Contain particles that mimic the pathogen structures but remain non-infectious.*											

4. **Please evaluate your level of fear related to COVID-19 vaccination using a 1-10 scale, where 1 – no fear, 5 – medium level of fear, 10 – a very high level of fear.**

   1 2 3 4 5 6 7 8 9 10

5. **What are your primary reasons behind a fear related to COVID-19 vaccination?** *(multiple answer question)*
   I have no fears
   The onset of serious allergic reaction following the vaccine administration (e.g., anaphylaxis)
   The onset of other severe adverse effects following the vaccine administration
   Potential unknown long-term adverse effects of the vaccine
   Potential adverse effect on fertility
   Potential induction of autoimmune reactions following the vaccine administration
   Other, please specify:...

6. **Please evaluate your level of trust in the COVID-19 vaccines authorized for use in Poland using a 1-10 scale, where 1 – no trust, 5- medium level of trust, 10 – high level of trust.**

**Vaccine**	**1**	**2**	**3**	**4**	**5**	**6**	**7**	**8**	**9**	**10**
**mRNA vaccine BioNTech/Pfizer** (BNT162b2)										
**mRNA vaccine Moderna** (mRNA-1273)										
**Vector vaccine AstraZeneca** (AZD1222)										

7. **Please evaluate your level of trust in the COVID-19 vaccines not authorized for use in Poland using a 1-10 scale, where 0 – I have not heard about this vaccine type, 1 – no trust, 5- medium level of trust, 10 – high level of trust.**

**Vaccine**	0	1	2	3	4	5	6	7	8	9	10
**Vector vaccine Sputnik V**											
**Vector vaccine Johnson & Johnson** (Ad26.COV2.S)											
**mRNA vaccine CureVac** (CVnCoV)											
**Protein vaccine Novavax** (NVX-CoV2373)											
**Inactivated vaccine Sinovac** (CoronaVac)											
**Inactivated vaccine Dinopharm** (BBIBP-CorV)											
**Inactivated vaccine Covaxin**											
**Vector vaccine Cansino** (Ad5-nCoV)											

8. What are your primary reasons behind the willingness to receive a COVID-19 vaccine?
(multiple answer question)
Protecting myself from infection
Protecting relatives from infection
Putting an end to the pandemic
Other, please specify:...

9. **Are you a COVID-19 convalescent individual?** Yes No
10. **Did you experience the death of your relative due to COVID-19?** Yes No
11. What are your primary sources of information on COVID-19 vaccines?
(multiple answer question)
I do not seek information on COVID-19 vaccines
TV
Radio
Press
Expert materials
Scientific literature
Information from relatives/friends
12. **Your age** (in years):...
13. Your place of living:
Rural
Urban ≥250,000 residents
Urban 50,000–200,000 residents
Urban 50,000–100,000 residents
Urban < 50,000 residents
14. **Your gender:** Female Male
15. Your education level:
Primary
Secondary
Vocational
Tertiary
16. **If tertiary, what is your field of discipline?** (For assistance, see the Polish classification of scientific fields; https://pl.wikipedia.org/wiki/Klasyfikacja_dziedzin_i_dyscyplin_naukowych_w_Polsce)
Medicine and health
Exact and natural science
Agricultural
Technology
humanities
Social
Theology
Art
